# Transcatheter aortic valve replacement using the iSleeve expandable sheath in small femoral arteries

**DOI:** 10.1136/openhrt-2021-001703

**Published:** 2021-10-12

**Authors:** Natalie Glaser, Crochan J. O'Sullivan, Nawzad Saleh, Dinos Verouhis, Magnus Settergren, Rickard Linder, Andreas Rück

**Affiliations:** 1 Department of Molecular Medicine and Surgery, Karolinska Institutet, Stockholm, Sweden; 2 Department of Cardiology, Stockholm South General Hospital, Stockholm, Sweden; 3 Department of Cardiology, Bon Secours Hospital, Cork, Ireland; 4 Department of Cardiology, Karolinska University Hospital, Stockholm, Sweden; 5 Department of Medicine, Karolinska Institutet, Stockholm, Sweden

**Keywords:** heart valve prosthesis implantation, transcatheter aortic valve replacement, epidemiology, aortic valve stenosis

## Abstract

**Background:**

Small femoral arteries have been associated with a higher risk of vascular complications in transfemoral transcatheter aortic valve replacement (TAVR). We investigated the feasibility and safety of TAVR in patients with small femoral arteries.

**Methods:**

In this observational study, we included 82 patients who underwent transfemoral TAVR with the ACURATE neo system using the expandable 14F iSleeve sheath between 2018 and 2019 at Karolinska University Hospital, Sweden. Of these, 41 patients had a minimal femoral artery diameter of ≥5.5 mm (mean 6.5, range 5.5–9.2), and 41 patients had a minimal femoral artery diameter <5.5 mm (mean 4.9, range 3.9–5.4).

**Results:**

There was no significant difference in major vascular and bleeding complications between the small femoral artery group (7%) and the normal femoral artery group (2%) (p=0.62). The total of major and minor vascular complications did not differ significantly according to femoral artery size (17% vs 5%) (p=0.16). The iSleeve sheath was not correlated with any of the complications. The use of the iSleeve sheath was unsuccessful in four patients (5%), of which one patient had a small femoral artery diameter.

**Conclusion:**

Transfemoral TAVR with the ACURATE neo system using the iSleeve sheath is a promising method for patients with small femoral arteries even though we found a trend towards higher rates of complications in these patients. The use of expandable sheaths may expand the spectrum of patients that can be treated with transfemoral TAVR, and thus may improve the prognosis in patients with severe aortic valve stenosis.

**Key questions**

**What is already known about this subject?**

- Small femoral arteries have been associated with a higher risk of vascular complications in transfemoral transcatheter aortic valve replacement (TAVR).

**What does this study add?**

- This study adds information about the feasibility and safety with TAVR using an expandable sheath in patients with small femoral arteries.

**How might this impact on clinical practice?**

- Transfemoral TAVR with the ACURATE neo system using the iSleeve sheath is a promising method for patients with small femoral arteries even though we found a trend towards higher rates of complications in these patients. The use of expandable sheaths may expand the spectrum of patients that can be treated with transfemoral TAVR and thus may improve the prognosis in patients with severe aortic valve stenosis.

## Introduction

The use of transcatheter aortic valve replacement (TAVR) for treatment in patients with severe aortic valve stenosis has seen a rapid increase during the last decade. In TAVR, transfemoral access is associated with better outcomes compared with alternative routes of access.^1^ One of the main limitations with transfemoral TAVR is the risk of major vascular complications, which occur in 4.5%–8.0% of the patients.^2 3^ Vascular complications have been associated with increased risk of bleeding, need for blood transfusion and mortality.^4 5^ Newer-generation transfemoral TAVR system sheaths are indicated for use in patients with femoral artery diameters of at least 5.5 mm, and approximately 10% of patients cannot undergo transfemoral TAVR.^6^ Small femoral artery diameters have been associated with a higher risk of vascular complications^7 8^ and are one of the reasons to choose alternative access routes. Expandable sheaths that allow a continuous expansion as the valve is advanced through the sheath have been developed to reduce vascular complications and to facilitate transfemoral access. Previous studies have shown that smaller sheath diameters can contribute to lower rates of vascular complications.^9 10^ Therefore, we performed a study using the expandable 14F iSleeve sheath (Boston Scientific, Marlborough, Massachusetts, USA) to analyse feasibility and safety of transfemoral TAVR in patients with femoral artery diameters below 5.5 mm.

## Patients and methods

### Study design and study population

Methods and data analysis were reported according to guidelines.^11^ We included 41 consecutive patients with severe aortic stenosis and a femoral artery diameter of ≥5.5 mm and 41 consecutive patients with a femoral artery diameter of <5.5 mm who underwent transfemoral TAVR with the self-expandable ACURATE neo (Boston Scientific) system at Karolinska University Hospital, Stockholm, Sweden, between March 2018 and February 2019. All patients who are evaluated for TAVR at Karolinska University Hospital undergo multidetector computed tomography (MDCT) angiography before discussion within the multidisciplinary heart team, which makes the final decision to plan the patient for transfemoral TAVR. The percentage of transfemoral access was high (98.1%) for all TAVR procedures during the study period at our institution.

The inclusion of patients started in March 2018 when the iSleeve sheath became available in a limited market release. The preprocedural MDCT was evaluated by the interventional cardiologists using the 3mensio software (Pie Medical Imaging BV, Maastricht, The Netherlands). All MDCT images were re-evaluated by the director of TAVR at Karolinska University Hospital, who is also one of the authors (AR), at the end of the study period. The MDCT evaluation included the minimal luminal diameter of the access artery (defined as the smallest minimal femoral or iliac artery diameter on the main access side), and a classification of tortuosity and calcification along the iliac arteries (none, mild, moderate and severe). Patients were divided into two groups: the normal femoral artery group (minimal luminal diameter ≥5.5 mm) and the small femoral artery group (minimal luminal diameter <5.5 mm). The sheath-to-femoral artery ratio (SFAR) was calculated by dividing the sheath’s outer diameter at the expandable section (7.3 mm) by the femoral artery minimal luminal diameter.

The iSleeve sheath has a 14F profile (4.8 mm) when not expanded and a 32 cm working length. It is compatible with the ACURATE neo system and CE marked since early 2018. Specifically, the iSleeve diameter is 7.3 mm at the expandable section when the valve is crossing the sheath, and 7.9 mm at the arteriotomy site. Four experienced invasive cardiologists who never used the iSleeve sheath before the start of this study performed all 82 TAVR procedures. Baseline and operative characteristics were gathered at the time of intervention, and data were completed retrospectively from medical records with information about outcomes. After all data were collected; data were anonymised; and data management and statistical analysis proceeded with fully anonymised data.

### Outcomes

The primary objective was feasibility and safety of TAVR with the ACURATE neo system in patients with small femoral artery diameters using the iSleeve expandable sheath. Feasibility was defined as the ability to successfully introduce the iSleeve sheath and to cross and retrieve through the sheath with predilation and postdilation balloons, without the need to change to another sheath. Safety was defined as in-hospital major or minor vascular complications, or major bleeding complications. These outcomes were evaluated according to the updated Valve Academic Research Consortium-2 criteria.^5^

### Procedural technique and details

The TAVR procedure performed at Karolinska University Hospital followed routine medical procedures and was performed with local anaesthesia in all patients, with fentanyl as analgesia when needed. Ultrasound-guided micropuncture was used for femoral access. The iSleeve sheath was introduced under fluoroscopic guiding. The introduction of the sheath was preceded by balloon dilation of the iliac artery in seven patients. Predilatation of the aortic stenosis was performed with a True balloon (Bard Peripheral Vascular, Tempe, Arizona, USA) at a size 1–2 mm smaller than the perimeter derived mean annular diameter. The transcatheter aortic valve prosthesis was implanted with standard techniques (moving image 1, online supplemental data). Intravenous unfractionated heparin was administered during the procedure with a target activated clotting time of 250 s. Protamine was used to reverse the heparin effect if deemed beneficial by the operator. The 18F MANTA device (Teleflex, Wayne, Pennsylvania, USA) was used for vascular closure. Postprocedural peripheral blood flow was checked with manual palpation and pulsed-wave ultrasound in the superficial femoral artery after each procedure. Light compression with a manual femoral compression device, Femostop (Abbott Vascular, Abbott Park, Illinois, USA), was used routinely for 3 hours postoperatively. The interventional cardiologist was responsible for selecting the optimal preoperative and postoperative antithrombotic regimen.

10.1136/openhrt-2021-001703.supp1 Supplementary video

### Statistical analysis

Baseline characteristics were described as means and SD for continuous variables and as numbers and proportions for categorical variables. Fisher’s exact test was used to statistically assess the rate of complications between small and normal femoral artery diameters. Stata V.16.1 was used for data management and statistical analysis.

## Results

### Baseline and operative characteristics

**Figure 1:**
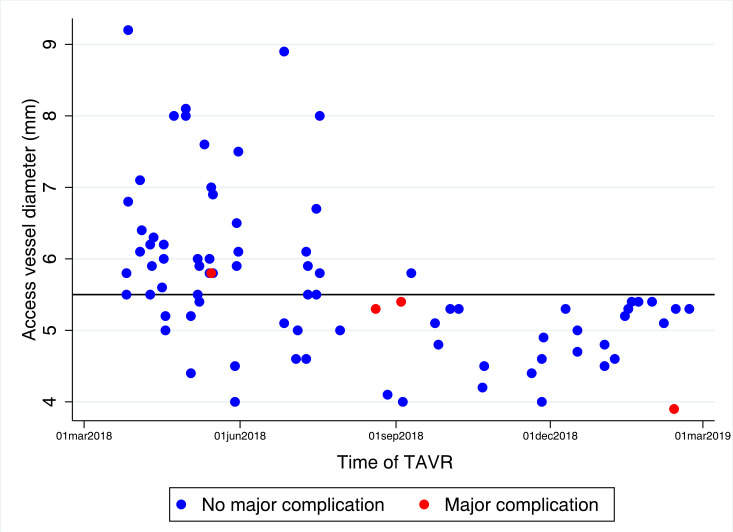
Femoral artery diameter and major vascular complications. Femoral artery diameters and occurrence of major vascular complications in 82 patients who underwent transfemoral TAVR with the ACURATE neo system using the iSleeve sheath at Karolinska University Hospital, Stockholm, Sweden, between 2018 and 2019. TAVR, transcatheter aortic valve replacement.

Among the 82 patients who were included in the study, the average femoral artery diameter was 5.7 mm in the total study cohort, 6.5 mm in the normal vessel group and 4.9 mm in the small vessel group (moving image 2, online supplemental data). Figure 1 illustrates the distribution of femoral artery diameters. The mean age was 81.7 (SD 6.6) years; 55% were women; and 35% had undergone previous percutaneous coronary intervention or coronary artery bypass grafting. The mean EuroSCORE II was 7.3 (SD 10.9), and the mean SFAR was 1.33 (SD 0.2). Patients in the small femoral artery group were younger and had a lower body mass index, a higher frequency of peripheral artery disease and a lower EuroSCORE II. Protamin was given to reverse the effect of the unfractionated heparin in 11 (27%) and 12 (29%) patients in the normal femoral artery group and the small femoral artery group, respectively. Balloon dilatation of the iliac artery was necessary to get the TAVR system through the sheath in eight patients in the small femoral artery group and in no patients in the normal femoral artery group. Baseline and operative characteristics according to femoral artery size are summarised in tables 1 and 2.

10.1136/openhrt-2021-001703.supp2 Supplementary video

**Table 1:** Baseline and operative characteristics in 82 patients who underwent transcatheter aortic valve replacement with the ACURATE neo system and the iSleeve sheath at Karolinska University Hospital, Stockholm, Sweden, between March 2018 and February 2019

	All patients N=82	Normal vessel diameter* N=41	Small vessel diameter* N=41
Age (years), mean (SD)	81.7 (6.6)	82.7 (6.7)	80.6 (6.4)
Female sex	45 (55)	22 (54)	23 (56)
Current smoker	5 (6)	0 (0)	5 (12)
Body mass index (kg/cm^2^), mean (SD)	25.5 (4.9)	26.1 (5.6)	24.8 (4.0)
Diabetes mellitus	19 (23)	10 (24)	9 (22)
Peripheral artery disease	12 (15)	4 (10)	8 (20)
Atrial fibrillation	29 (35)	13 (32)	16 (39)
Previous coronary artery bypass grafting	7 (9)	3 (7)	4 (10)
Previous percutaneous coronary intervention	21 (26)	11 (27)	10 (24)
Previous arterial intervention on same side	2 (2)	1 (2)	1 (2)
Previous arterial access on same side within 6 months	6 (7)	0 (0)	6 (15)
EuroSCORE II, mean (SD)	7.3 (10.9)	7.8 (14.6)	6.8 (5.8)
Minimal femoral artery diameter (mm), mean (SD)	5.7 (1.1)	6.5 (1.0)	4.9 (0.5)
Access vessel calcification†			
None	2 (2)	2 (5)	0 (0)
Mild	30 (37)	22 (54)	8 (20)
Moderate	39 (48)	16 (39)	23 (56)
Severe	11 (13)	1 (2)	10 (24)
Access vessel tortuosity†			
None	1 (1)	1 (2)	0 (0)
Mild	48 (59)	24 (59)	24 (59)
Moderate	32 (39)	15 (37)	17 (41)
Severe	1 (1)	1 (2)	0 (0)
Preoperative aortic valve mean pressure gradient, mean (SD)	47.9 (11.7)	49.5 (13.5)	46.4 (9.6)
Sheath-to-femoral artery ratio, mean (SD)	1.33 (0.2)	1.15 (0.2)	1.52 (0.2)

Data are n (%) unless otherwise noted.

*Normal vessel diameter defined as at least 5.5 mm and small vessel diameter as below 5.5 mm.

†Subjectively assessed on multidetector computed tomography.

**Table 2:** Operative characteristics in 82 patients who underwent transcatheter aortic valve replacement with the ACURATE neo system and the iSleeve sheath at Karolinska University Hospital, Stockholm, Sweden, between March 2018 and February 2019

	All patients N=82	Normal vessel diameter* N=41	Small vessel diameter* N=41
Heparin dose (IU), mean (SD)	5235 (1527)	5113 (1347)	5353 (1693)
Highest activated clotting time (seconds), mean (SD)	283 (49)	283 (51)	284 (44)
Protamin given	23 (28)	11 (27)	12 (29)
Second arterial access through radial artery	59 (72)	36 (88)	23 (56)
Valve size (mm)			
23	12 (15)	6 (15)	6 (15)
25	36 (44)	17 (43)	19 (46)
27	33 (41)	17 (43)	16 (39)

Data are n (%) unless otherwise noted.

*Normal vessel diameter defined as at least 5.5 mm and small vessel diameter below 5.5 mm.

### Feasibility and safety

There were no in-hospital deaths and the in-hospital data were complete for all patients. There was no significant difference in major vascular and bleeding complications between the small femoral artery group (7%) and the normal femoral artery group (2%) (p=0.62). Also, the total of major and minor vascular complications did not differ significantly according to femoral artery size (17% vs 5%) (p=0.16). The same four patients who had a major vascular complication also had major bleeding complications and, additionally, two had minor vascular complications. Minor vascular complications occurred in five additional patients. Consequently, any type of vascular or bleeding complication occurred in nine patients (11%). In these patients, an ACURATE neo valve size medium was implanted in seven patients, small in one patient and large in one patient. None of the complications that occurred were caused by the iSleeve sheath. For distribution of femoral artery diameters and the occurrence of major complications, see figure 1. There was no significant association between a sheath-to-femoral-artery ratio of >1.05 and higher complication rates (p=0.60).

Details on complications can be found in table 3. Of the patients with major complications, one patient had a dissection and thrombus distal to the puncture site that required aspiration and surgery. The same patient also had bleeding from the puncture site requiring blood transfusion. Two patients developed a pseudoaneurysm at the access site that was treated with thrombin injection, these patients also had major bleeding. One patient suffered occlusion of the femoral artery requiring vascular surgery and blood transfusion. Of the patients with minor complications, three patients suffered access site bleedings that were treated with endovascular stenting or balloon therapy. One patient suffered femoral occlusion that required unplanned endovascular balloon therapy. The seventh patient with minor vascular complication had a stenosis at the access site, which was treated with endovascular balloon at the end of the procedure. The use of the iSleeve sheath was unsuccessful in four patients (5%), of which three had a femoral artery diameter of at least 5.5 mm. One of these patients had a stent in the iliac artery (and a totally occluded iliac artery at the other side), which could not be passed with any sheath or valve prosthesis, despite balloon dilatation of the stent. In a second patient, the iSleeve sheath was damaged when the delivery system was retrieved and subsequently replaced with a 20F sheath. In the third patient, the iSleeve sheath kinked and was replaced with a 20F sheath; and in the fourth patient, the iSleeve sheath invaginated after valve delivery and was replaced with an 18F sheath.

**Table 3:** Detailed description of patients with complications according to the VARC criteria, and unsuccessful use of the iSleeve sheath

Patient	iSleeve unsuccessful	VARC major vascular complication	VARC major bleeding complication	VARC minor vascular complication	Age	Sex	Access vessel diameter	Calcification (0–3)	Tortuosity (0–3)	SFAR
1		Dissection and thrombus distal to puncture site, required aspiration and vascular surgery	Bleeding from access site requiring transfusion of two units of blood	–	87	Female	5.8	2	2	1.26
2		Pseudoaneurysm at access site leading to major bleeding	Bleeding at access site requiring transfusion of three units of blood	Pseudoaneurysm at access site	89	Female	5.4	3	1	1.35
3		Pseudoaneurysm at access site leading to major bleeding	Bleeding at access site with drop in haemoglobin level of 33.0 g/L	Pseudoaneurysm at access site	85	Male	5.3	2	1	1.38
4		Occlusion of femoral artery after MANTA, required vascular surgery	Bleeding after vascular surgery requiring transfusion of two units of blood	–	80	Female	3.9	3	2	1.87
5	–	–	–	Bleeding at access site with unplanned endovascular stenting	76	Female	4.1	2	1	1.78
6	–	–	–	Stenosis at access site with unplanned endovascular balloon therapy	74	Female	6	1	1	1.22
7	–	–	–	Bleeding at access site with unplanned endovascular stenting	86	Female	5	2	1	1.46
8	–	–	–	Bleeding at access site with unplanned endovascular balloon therapy	95	Female	4.9	2	1	1.49
9	–	–	–	Femoral occlusion with unplanned endovascular balloon therapy	71	Male	4.4	2	2	1.66
10	Could not pass stent in iliac artery with any sheath or valve prosthesis	-	–	–	88	Male	5.6	3	2	1.30
11	Damaged sheath				85	Female	5.5	1	1	1.33
12	Sheath kinked				81	Male	8	1	2	0.91
13	Invagination of iSleeve after valve delivery				85	Female	4.7	2	2	1.55

SFAR, sheath-to-femoral artery ratio; VARC, Valve Academic Research Consortium.

## Discussion

In this observational cohort study, we found that patients with small femoral arteries who underwent transfemoral TAVR with the ACURATE neo system using the expandable iSleeve sheath had an acceptable rate of major vascular and bleeding complications (7%). The mean femoral artery diameter in the small femoral artery group was 4.9 mm (range 3.9–5.4 mm), which is smaller than in previous studies.^7 8 12^ The small femoral artery group included eight patients where balloon dilatation of the iliac artery was necessary to get the TAVR system through the sheath. There was a trend towards more complications in patients with small compared with normal femoral artery diameters, but this association was not statistically significant and may be explained by the small sample size. Transfemoral TAVR using the expandable iSleeve sheath was feasible and safe in patients with both small and normal femoral artery diameters.

Although we could find no significant difference in vascular complications between normal and small size femoral arteries, there was a numerical trend towards more complications in patients with small femoral arteries, and it seems plausible that such an association exists. Some previous studies have shown an association between small access artery size and vascular complications.^7 8^ A smaller study by Abu Saleh *et al*, using a balloon-expandable and recollapsible 19F sheath, included 13 patients with a minimal luminal diameter of <5.0 mm and 51 patients with larger arteries, and found no major vascular complications at all.^12^ In the study by Abu Saleh *et al*, a balloon-expandable and recollapsible 19F sheath was used. Compared with this study,^12^ the patients in our study had smaller artery diameters, and the study sample was larger.

Expandable sheaths, like the 14F iSleeve, have one or more longitudinal slits with thinner material where the sheath is able to expand. However, this decreases the longitudinal strength of the sheath, which might explain why the iSleeve sheath was kinked either during insertion or after retrieval of the predilatation balloon in three patients in our study cohort. Of note, the iSleeve has undergone a slight redesign subsequently.

Previous studies showed that an SFAR of >1.05 was associated with an increased risk of vascular complications.^9 13 14^ However, other studies could not confirm this association.^15 16^ In our study, no association between higher SFAR and rate of vascular complications could be found. Our results strengthen the results previously reported that SFAR is not a predictor of vascular complications. Furthermore, most complications occurred in patients who received an ACURATE neo valve size medium. However, these results have to be interpreted with caution due to the small number of events in our study.

As TAVR with non-femoral access has been associated with adverse outcomes,^1^ it would be valuable to find ways to enable transfemoral access also in patients who have previously been considered ineligible for transfemoral access. For example, the use of intravascular lithotripsy could be one way to facilitate transfemoral access.^17^ In the present study, we used an expandable sheath to facilitate transfemoral access. In our opinion, a 7% rate of major vascular complications but no mortality in patients with small sized femoral arteries is acceptable, considering the alternative with non-femoral access and the associated risks.

### Strengths and limitations

The number of patients included and the events recorded in our study were limited, and therefore, it is possible that a significant difference in the rate of complications between patients with normal and small access vessels could not be detected. Furthermore, we did not include a control group that underwent TAVR with a traditional sheath during the same time period. Thus, the results of this study should be regarded as merely hypothesis-generating in terms of the feasibility of transfemoral TAVR with the iSleeve sheath. Also, the incidence of vascular complications is largely affected by the puncture and closure technique used. We used an ultrasound-guided micropuncture technique and the MANTA device for vascular closure in all patients. Thus, our results may not be generalisable to settings with other puncture and closure techniques. A strength of this study is that we included 82 consecutive patients who underwent transfemoral TAVR, which increases the generalisability and the internal validity. Other strengths include the complete follow-up for all outcomes and the use of MDCT in all patients for evaluation of the minimum femoral artery diameter.

## Conclusion

Transfemoral TAVR with the ACURATE neo system using the expandable iSleeve sheath is a promising method for patients with small femoral arteries. However, there was a numerical trend towards higher rates of complications in these patients, and our results should be confirmed in larger study populations. The use of expandable sheaths may expand the spectrum of patients that can be treated with transfemoral TAVR and thus improve the prognosis in patients with severe aortic valve stenosis.

## Data Availability

All data relevant to the study are included in the article or uploaded as supplementary information.
